# Livestock-Associated Methicillin-Resistant *Staphylococcus aureus* From Animals and Animal Products in the UK

**DOI:** 10.3389/fmicb.2019.02136

**Published:** 2019-09-12

**Authors:** Muna F. Anjum, Francisco Marco-Jimenez, Daisy Duncan, Clara Marín, Richard P. Smith, Sarah J. Evans

**Affiliations:** ^1^Department of Bacteriology, Animal and Plant Health Agency, Weybridge, United Kingdom; ^2^Instituto de Ciencia y Tecnología Animal, Universitat Politècnica de València, Valencia, Spain; ^3^Department of Epidemiological Sciences, Animal and Plant Health Agency, Weybridge, United Kingdom; ^4^Departamento de Producción Animal, Sanidad Animal, Salud Pública Veterinaria y Ciencia y Tecnología de los Alimentos, Facultad de Veterinaria, Instituto de Ciencias Biomédicas, Universidad CEU Cardenal Herrera, CEU Universities, Valencia, Spain

**Keywords:** MRSA, United Kingdom, livestock, animal products, animal

## Abstract

Livestock-associated methicillin-resistant *Staphylococcus aureus* (LA-MRSA) is an emerging problem in many parts of the world. Although animal-adapted LA-MRSA has been known for many years, recent reports suggest a possible increasing trend in the zoonotic transmission of LA-MRSA in Europe. Since its emergence in the early 2000’s, several investigations have indicated that persons in prolonged, repeated contact with affected livestock are at a higher risk of becoming colonized with LA-MRSA. LA-MRSA monitoring in livestock is voluntary under current EU legislation, and not all member states, including the UK, participate. UK LA-MRSA isolates have been detected through scanning surveillance, where samples are submitted from clinically diseased livestock for diagnostic investigation, and research studies. Surveys conducted on retail beef, pig and poultry meat on sale in the UK have also detected LA-MRSA. Taken together these results suggest that LA-MRSA is present in the UK, possibly at low prevalence level, as suggested by available evidence. In this review, we examine the data available from UK livestock and animal products, and make recommendations for future. We also review the findings from whole genome sequencing (WGS) of the possible lineage of some UK livestock isolates.

## Introduction

Livestock associated MRSA (LA-MRSA) was first described in 2005 (Voss et al., 2005) where a new clone of MRSA of sequence type (ST) 398 was identified and grouped within clonal complex (CC) 398^1^. The LA-MRSA CC398 lineage, which apparently emerged in European pigs between 2003 and 2005, has since detected in other animal species in many European countries and also in North America, where it can colonize the animal but only rarely cause infections (Voss et al., 2005; Khanna et al., 2008; Smith et al., 2009, 2013; Goerge et al., 2017). Asymptomatic colonization is common although it can cause a variety of human and animal infections including fatal courses, as described for S. *aureus* and MRSA where heavily colonized carriers are more likely to be infected than transient or intermittent carriers (Bradley, 2007; Cuny et al., 2015). However, the human disease burden of LA-MRSA is lower compared to other MRSA lineages possibly because patients affected by MRSA CC398 generally show different demographics in that they are younger, or stay for a shorter time in hospital, and the clinical characteristics are usually less severe or complicated (Becker et al., 2017); but LA-MRSA CC398 is thought not to be inherently less pathogenic for humans than *S. aureus* (Cuny et al., 2013). In fact, LA-MRSA is an emerging category of *S*. *aureus* throughout the world (Smith, 2015).

Several studies have speculated that CC398 MSSA originated in humans but lost human associated factors such as Panton-Valentine Leukocidin (PVL)-associated phages, toxic shock syndrome toxin I and exfoliative toxins, which are markers of community associated (CA)-MRSA and hospital associated (HA)-MRSA strains (Kadlec et al., 2012; Mohamed et al., 2012; Ballhausen et al., 2017), and acquired antibiotic resistance genes such as *mecA* and *tetM* as they adapted to livestock (Fitzgerald, 2012a, b; Price et al., 2012). Furthermore, several investigations have shown that persons in contact with livestock may be at increased risk of becoming colonized with LA-MRSA. LA-MRSA CC398 colonization has been detected in 24–86% of pig-, 31–37% of cattle-, and 9–37% of poultry-farmers, as well as 44–45% of pig-care veterinarians in European countries (Goerge et al., 2017). Nevertheless, colonization is thought to be dependent on frequency and intensity of animal contact and the duration of exposure, as livestock are thought to be transiently rather than permanently colonized (Bangerter et al., 2016) Also, human contamination of carcasses or meat product at abattoir or meat processing plants may occur and be a source of MRSA, which are not livestock associated (Hadjirin et al., 2015).

Although CC398 is the main lineage associated with MRSA isolated from livestock other clonal complexes, and sequence types (STs) which are not within CC398, have also been associated with livestock and animal products, both in the UK and elsewhere. In this review we focus on the findings from UK livestock and animal products.

## LA-MRSA in the United Kingdom

During the last decade, LA-MRSA (mainly MRSA CC398), has become increasingly common among pigs in several European countries (Verkade and Kluytmans, 2014); it has also been reported from humans and animal products (European Food Safety Authority, and European Centre for Disease Prevention, and Control, 2015). Surveillance for LA-MRSA in animals and food is voluntary in the European Union, although the European Food Safety Authority (EFSA) recommends routine surveillance for LA-MRSA in broiler flocks, fattening pigs and dairy cattle. The recommendation also includes veal calves under 1 year of age and fattening turkey flocks in countries where production exceeds 10 million tonnes slaughtered/year. Prevalence data for LA-MRSA is available from all member states that participate in the routine systematic surveillance in annual reports compiled by EFSA (European Food Safety Authority and European Centre for Disease Prevention, and Control, 2017). However, the production levels in the UK for veal calves are below these thresholds and there is currently no structured surveillance of LA MRSA in UK livestock. Despite increasing reports of LA-MRSA from animals and food sampled in continental Europe, there have been limited reports of its recovery in the UK (Table 1).

**TABLE 1:** Published reports of LA-MRSA from livestock and animal-derived food products in the United Kingdom.

**Type**	**Clonal information**	**Species**	**Studies**
Farm	ST398	Pig	Hartley et al., 2014
Farm	ST398	Pig	Hall et al., 2015
Farm	ST398	Pig	Sharma et al., 2016
Farm	CC30	Pig	Lahuerta-Marin et al., 2016
Farm	CC398	Various	Anonymous, 2017^∗^
Farm	ST398	Turkey	GOV. UK, 2013
Farm	CC9/CC398	Turkey	Sharma et al., 2018
Game Farm	CC9/CC398	Pheasant	Sharma et al., 2018
Farm	CC398	Beef cattle calf	Stone, 2017
Meat	CC9, CC22	Chicken, pork and beef	Dhup et al., 2015
Meat	ST398	Pork and chicken	Hadjirin et al., 2015
Meat	ST398	Chicken, turkey and pork	Fox et al., 2017
Milk bulk tank	CC130, CC705, ST425	Dairy cattle	Garcia-Alvarez et al., 2011
Milk bulk tank	CC398	Dairy cattle	Paterson et al., 2012
Milk bulk tank	CC130, ST398, ST425	Dairy cattle	Paterson et al., 2014
Equine hospital	ST398	Horse	Loeffler et al., 2009
Equine hospital	CC398	Horse	Bortolami et al., 2017

*^∗^Provides summary of all LA-MRSA isolations from UK government laboratories since February 2013, including some isolates already listed in this table, which have been referenced separately e.g., CC30.*

A single structured prevalence study for LA-MRSA in pig herds was performed in the UK in 2008 as part of a wider study to determine the prevalence of positive pig herds in EU Member States (MS). The prevalence in MS ranged from 0 to 46% in breeding herds, and 0 to 51% in production/fattening herds (European Food Safety Authority, 2009). In the UK, a total of 258 pig holdings were tested and none was found positive for LA-MRSA CC398. This yielded 95% confidence intervals of 0.0–3.8% estimated prevalence in UK pig breeding herds and 0.0–1.8% in UK pig production herds (European Food Safety Authority, 2009). Following the 2008 study there have been sporadic reports of LA-MRSA in UK livestock although no further prevalence studies have been carried out. The majority of LA-MRSA detections have been made through scanning surveillance activities. Scanning surveillance comprises the submission of clinical samples from livestock, by veterinarians, to government laboratories, where microbiological and other investigations are performed to identify the possible causative agent. Scanning surveillance and other studies have reported the presence in the UK of LA-MRSA CC398, CC9, CC9/CC398 hybrid, CC22, CC30, CC130, CC705, and ST425 (Table 1). These isolates have been reported from horses (Loeffler et al., 2009; Bortolami et al., 2017), dairy cattle (Garcia-Alvarez et al., 2011; Paterson et al., 2012, 2014) beef cattle (Stone, 2017), poultry and pheasant (GOV. UK, 2013; Stone, 2017; Sharma et al., 2018), pigs (Hartley et al., 2014; Hall et al., 2015; Lahuerta-Marin et al., 2016; Sharma et al., 2016), pork meat (Dhup et al., 2015; Hadjirin et al., 2015; Fox et al., 2017), beef meat (Dhup et al., 2015), chicken and turkey meat (Dhup et al., 2015; Fox et al., 2017).

Government institutes involved in scanning surveillance includes the Animal and Plant Health Agency (APHA) in England and Wales, Agri-Food and Biosciences Institute (AFBI; Northern Ireland) and Scotland’s Rural College (SRUC). Generally *S. aureus* isolated by routine microbiology from diagnostic samples such as mastitis in cattle are tested for penicillin or ampicillin sensitivity by disc diffusion; only some pig and poultry samples are tested. Any penicillin/ampicillin resistant isolate is tested for cefoxitin and oxacillin antibiotic sensitivity for identification of presumptive *mecA* or *mecC* harboring MRSA. For any phenotypically positive isolates, a multiplex PCR that can detect *mecA*, as well as *S. aureus* species specific genes *nuc* and 16S rRNA (European Union Reference Laboratory for Antimicrobial Resistance, 2009) are used. For *mecA* negative samples a PCR is used for amplification of *mecA* and *mecC*, identification of *S. aureus* by amplification of the *spa* gene, and detection of the Panton-Valentine Leukocidin (PVL or LukF PV) encoding gene (Stegger et al., 2012). In any *mecA* or *mecC* positive sample, *spa*-typing and WGS is performed (Sharma et al., 2016, 2018). Between 2013 and 2015, nine LA-MRSA were identified after screening more than 1000 *S. aureus* isolated from diagnostic submissions submitted through scanning surveillance to APHA and AFBI (160); none had been reported from Scotland during this period. A low frequency of detection has continued since 2015. Details of the UK isolates gathered from scanning surveillance and other studies are provided in Table 1. The disease surveillance and microbiological laboratories based in Northern Ireland and Scotland are involved in isolation of LA-MRSA from these countries.

## Background of UK LA-MRSA Isolates

### Isolation From Scanning Surveillance of Livestock

The first confirmation of CC398 LA-MRSA on a pig farm in the UK was reported in 2014, and the piglet harboring CC398 LA-MRSA was one of a group of five piglets submitted to the Omagh disease surveillance laboratory, AFBI, with a history of pneumonia and wasting (Hartley et al., 2014). Also in 2014, CC398 LA-MRSA was isolated from one 10-day-old piglet with skin lesions submitted to an APHA veterinary investigation center (Hall et al., 2015). CC398 LA-MRSA has been isolated from the caecal content of healthy pigs at abattoir from 2 of 56 pig farms in England that were sampled during 2014–2015, as part of a research project based at APHA (AbuOun et al., 2017). All three APHA isolates belonged to *spa*-type t011 and showed similar characteristics to other UK and European CC398 LA-MRSA strains by WGS (Sharma et al., 2016). In 2015, three pigs with signs of ill-thrift (low rate of growth) from a farm in Northern Ireland were submitted to the AFBI Veterinary Sciences Division for post-mortem investigation. *S. aureus* was obtained from different tissues in the three animals and all nine detected *S. aureus* isolates were identified as CC30 MRSA (Lahuerta-Marin et al., 2016). The APHA also reported detection of LA-MRSA CC398 in 2016 through scanning surveillance from pigs during a non-clinical investigation, and in 2017 from pigs during a clinical investigation (Anonymous, 2017). In addition, CC398 LA-MRSA, isolated from seven clinical investigations in pigs from Northern Ireland, have been reported between 2014 and 2017 (Anonymous, 2017).

The first isolation of CC398 LA-MRSA at farm level was reported in November 2013 in turkeys on a poultry farm (GOV. UK, 2013), where all isolates were shown to belong to *spa*-type t011 with WGS indicating the same clone to be disseminated across the farm (Sharma et al., 2016). LA-MRSA CC398 isolates were reported by APHA from UK fattening turkeys in 2016 from England. The turkey was incidentally diagnosed with LA-MRSA CC398 *spa*-type t899 while under investigation for an unrelated upper respiratory tract infection (Stone, 2017). A CC398 LA-MRSA, also of *spa*-type t899, isolated from a pheasant during a clinical investigation in Scotland was reported in 2017 (Anonymous, 2017). Both avian isolates were shown by WGS to belong to the CC9/C398 hybrid genotype (Sharma et al., 2018).

Government surveillance reported LA-MRSA CC398 isolates detected by APHA from a spontaneously aborted calf from England/Wales in 2016, and from a single clinical investigation of cattle from Northern Ireland between 2014 and 2017 (Anonymous, 2017; Stone, 2017).

### Isolation From Retail Meat

Three point-prevalence surveys were conducted on meat products by Dhup in 2011, and Hadjin and Fox in 2015, at UK retail outlets; all three studies examined pork meat. CC398 LA-MRSA was identified in 3 of 52 (5.8%) (Hadjirin et al., 2015) and 3 of 63 (4.7%) pork products (Fox et al., 2017) on sale in 2015, while CC9 LA-MRSA was identified in 1 of 30 (3.3%) pork products on sale in 2011 (Dhup et al., 2015). It is noteworthy that only one of the three products from the Fox et al. study (Fox et al., 2017) was specified as UK origin.

Although LA-MRSA has not been detected directly on UK broiler farms, two out of three studies that examined chicken meat at retail isolated LA-MRSA. In 2011, CC22 a common HA-MRSA, was reported from 2 of 30 samples (6.7%) suggesting contamination from human sources; while one CC9 isolate (3.3%) that lacked the immune evasion cluster characteristic of LA-MRSA, was detected (Dhup et al., 2015). CC398 LA-MRSA was also reported in four of 50 (8.0%) chicken meat samples and two of 11 (18.2%) turkey meat samples in 2015; although these samples were bought from retail meat outlets in North West England only three were from UK, two were from continental Europe, the origin of one was not specified (Fox et al., 2017).

In beef, CC22 MRSA was identified in 2011 from 1 of 30 meat samples (3.3%), suggesting contamination from human source during processing (Dhup et al., 2015).

When results from the 2011 retail study by Dhup et al. (2015) was compared with that from 2015 by Fox et al. (2017), which was performed in the same geographic region, it indicated a possible increase in prevalence of LA-MRSA in the UK. The authors suggested this could be due to the fact that 60% of meat consumed in the UK is imported from European countries where CC398 contamination has been reported in up to 60% of samples (Fessler et al., 2011; European Food Safety Authority, and European Centre for Disease Prevention, and Control, 2015; Dhup et al., 2015).

### Isolation From Milk

In bulk milk from dairy cattle, the first isolation of CC398 LA-MRSA was reported in 2012 (Paterson et al., 2012). A survey of 1500 bulk tank milk (BTM) samples from about 1500 farms was undertaken to determine the prevalence of both *mecA* and *mecC* MRSA. Seven *mecA* CC398 isolates were identified, including three from the same farm. A total of five geographically dispersed farms in the UK were positive for LA-MRSA CC398 (Shore et al., 2012). Following the detection and reporting of *mecC* MRSA from dairy cattle in England (Garcia-Alvarez et al., 2011), the same authors conducted a study to determine the occurrence of *mecC* MRSA in bovine bulk milk in Great Britain. A total of 1090 dairy farms were evaluated for the presence of *mecC* MRSA in BTM samples (Paterson et al., 2014). *mecC* MRSA was identified in 10 of 465 dairy farms (2.15%, [95% CI 1.17–3.91]) from England and Wales but not from 625 farms sampled from Scotland. However, only one CC398 LA-MRSA was identified (0.06%) from a farm in England. Three of the ten *mecC* MRSA isolates were CC130 and seven were ST425 (Paterson et al., 2014).

### Isolation From Horses

In 2009, CC398 LA-MRSA was reported from two horses (one with a history of travel outside UK) detected in a screening study at an equine hospital performed by the Royal Veterinary College (Loeffler et al., 2009). This was the first isolation of CC398 LA-MRSA reported from UK animals. Further analysis of these two isolates by WGS indicated that they resembled other *spa* type 11 strains, clustering closely to another horse isolate from Belgium (Sharma et al., 2016). In a study from University of Liverpool, presence of LA-MRSA CC398 isolates, predominantly of *spa*-type 11 was reported from surveillance at an UK Equine Veterinary Hospital from 2011 to 2016; 65 of the 829 samples collected from environmental sites, surgical site implants and hand-plates were CC398 (Bortolami et al., 2017).

## Whole Genome Sequencing of UK Livestock Isolates

MRSA lineages can be identified with molecular tests such as pulsed-field gel electrophoresis (PFGE), *Staphylococcus* protein A or *spa* typing or multiple locus variable number tandem repeat analysis (MLVA), multilocus sequence typing (MLST), and *Staphylococcal* cassette chromosome (*SCC*mec) typing. DNA microarrays, which have been used for determining virulence or antimicrobial gene presence in diverse bacteria (Hopkins et al., 2007; Carter et al., 2008; Wragg et al., 2009), have also been applied to MRSA, as have WGS (Schouls et al., 2009; Monecke et al., 2011; Piccinini et al., 2012; Shore et al., 2012; Sharma et al., 2016; Sabat et al., 2017). The latter has been particularly useful and is increasingly being used instead of PFGE for purposes such as tracing outbreaks, as well as identifying the most likely source of acquisition, which is an essential component of an effective surveillance system to describe epidemiological trends and infection control strategies (Rebic et al., 2016; Sharma et al., 2016). In addition, although phenotypic methods are easier to perform and interpret, and they are cost effective and widely available, they may be less discriminatory. Genotypic methods, although more expensive and technically demanding, can provide more detailed characterisation of the isolate (Anjum, 2015; Rebic et al., 2016). Specifically, WGS is a more rapid method for identifying genetic determinants such as virulence and AMR genes, as well as studying phylogenetic relationships between groups of isolates based on the core genome of hundreds of genes, rather than DNA microarray. The latter is based on a finite panel of selected genes and it can be time consuming to update with new genes; also it is becoming easier to apply WGS routinely as the associated costs are being reduced and bioinformatic tools are becoming more readily available (Anjum, 2015; Anjum et al., 2017).

Phylogenetic comparison of WGS data derived from a subset of the LA-MRSA CC398 isolates from livestock detected in United Kingdom, with isolates across Europe and North America, has indicated a possible European origin of UK LA-MRSA isolates. All UK isolates included in the phylogenetic comparison belonged to *spa-*types t011 and t034 (Sharma et al., 2016). Also, these isolates showed a multi-drug resistance genotype i.e., presence of three or more antimicrobial resistance genes including the tetracycline resistance gene, which is often present in LA-MRSA isolated from animals (Sharma et al., 2016). They did not harbor any human associated virulence factors such as the human φSa3 Immune Evasion Cluster (IEC) which contains a number of genes including staphylococcal complement inhibitor, staphylokinase and toxins that promotes survival within the human host (Xu et al., 2014); nor did they harbor the avian prophage carrying the SAAV_2008 and SAAV_2009 genes which have been detected in CC398 isolates from poultry, and produces resistance to killing by avian phagocytes (Argudin et al., 2013). However, as many of the isolates were from diseased livestock, further exploration of the WGS data is warranted to help identify possible animal-associated virulence factors, which may be another marker of adaptation in animals. A study which used genome-wide high-throughput screening to identify essential genes for CC398 LA-MRSA survival identified 24 genes important for survival in porcine blood; none of the genes were directly related to virulence factors (Christiansen et al., 2014).

More recently WGS of two *spa*-type t899 isolates, reported as incidental findings from a turkey and a pheasant in the UK, was performed. Both avian isolates harbored the *sac scn chp* genes associated with the φSa3 Immune Evasion Cluster. Also, both isolates were multi-drug resistant, although the heavy metal resistance gene *czrC*, encoding zinc resistance, was not detected in either isolate (Sharma et al., 2018). This is in contrast to previous findings where all nine UK isolates from pigs, cattle and turkey examined by WGS harbored the *czrC* gene (Sharma et al., 2016). Also, neither isolate harbored the avian prophage genes. Phylogenetic reconstruction indicated both isolates clustered with other *spa*-type t899 isolates, which display a unique genotype of being a CC9/CC398 hybrid. The English t899 turkey strain was closely related to t899 isolated from turkey in Germany and France, and from UK retail chicken meat, which had previously been classed as CC398 (Fox et al., 2017). The Scottish pheasant isolate, although still within the same phylogenetic cluster, was more distantly related (Sharma et al., 2018).

WGS has also been applied to isolates recovered from three pigs with signs of ill-thrift from a farm in Northern Ireland and shown to be phenotypically penicillin, cefoxitin and tetracycline resistant. The eight isolates identified as MRSA belonged to a novel CC30 clone, being positive for *lukM* and *lukF-P83* genes, a marker for virulence restricted to animal lineages which has been implicated in the pathogenesis of mastitis cattle and exudative dermatitis in squirrels (Lahuerta-Marin et al., 2016).

## Discussion

The fact that in the UK only a handful of LA-MRSA were identified after screening more than 1000 *S. aureus* isolated from diagnostic submissions from livestock submitted through scanning surveillance between 2013 and 2015 (Sharma et al., 2016), indicates it may be an emerging problem with currently a possible low prevalence. In some cases, LA MRSA was reported as an incidental finding and there was little evidence of it causing significant disease in animals. However, scanning surveillance is biased to investigations of clinically significant *S. aureus* isolates, which have originated primarily from cattle, with few isolates from pigs or poultry, where it may be present asymptomatically in the healthy population. In specific screening programs of pigs, MRSA is most commonly sampled through collection of nasal swabs; skin swabs can also be taken and sampling both skin behind the ear in conjunction with nasal swabs has been shown to be the most sensitive method for detection of MRSA in live pigs, suggesting a targeted approach to sampling is required (Pletinckx et al., 2012; Agerso et al., 2014). Furthermore, pooling of swabs can increase the sensitivity of detection of MRSA in herds compared to single swabs (Friese et al., 2012). Also, the MRSA status in individual pigs from a MRSA-positive herd can change, as pigs may be transiently rather than permanently colonized (Bangerter et al., 2016).

Although the frequency of reports in livestock in the UK remains low, the geographic and species dispersal suggests a widening distribution. Given the lack of available data, there is a need for surveillance of healthy livestock to establish how prevalent LA-MRSA is in UK livestock, and what the risks are for humans. The guidelines for harmonization of sampling strategies, outlined by EFSA, provide valuable guidance for the effective detection of LA-MRSA within the livestock industry (European Food Safety Authority, 2012).

Livestock-associated methicillin-resistant *Staphylococcus aureus* was also reported at a low-to-moderate prevalence in UK animal products such as bulk tank milk and retail meat, although the latter may partly reflect EU meat. It is also noteworthy that phylogenetic comparisons of UK livestock isolates through WGS, suggests a possible European origin with multiple incursions (Sharma et al., 2016, 2018).

Nevertheless, although frequency has increased since 2007, and CC398 is the dominant type, LA MRSA is rarely identified from human samples in the EU (Kinross et al., 2017). A recent assessment by the Food Standards Agency also reported a very low risk in the UK food chain and noted that there have been no reported foodborne outbreaks worldwide^2^. The need to revisit the assessment in the light of new data was recognized due to uncertainties on the prevalence of LA-MRSA in food and livestock. Furthermore, evidence from EU countries suggests a risk of transmission to people in contact with livestock but the zoonotic risk remains to be fully explored. The current lack of information suggests the need for systematic surveillance to understand reservoirs and transmission routes, applying a One Health approach and MRSA typing across sectors.

Harmonized structured surveillance would reduce uncertainties in risk assessment and linked epidemiological studies could be used to inform options for control and cost effectiveness. Furthermore, surveillance can be used to monitor the effectiveness of risk management at national and international levels as has been performed for zoonoses such as *Salmonella*.

## Author Contributions

MA and SE conceived the study. All authors wrote the manuscript.

## Conflict of Interest Statement

The authors declare that the research was conducted in the absence of any commercial or financial relationships that could be construed as a potential conflict of interest.

## Footnotes

1 http://saureus.mlst.net

2 https://pdfs.semanticscholar.org/4fca/b9277652e3d8cc34a8150b31de865a838e67.pdf
